# Predictive potential of preoperative Naples prognostic score-based nomogram model for the prognosis in surgical resected thoracic esophageal squamous cell carcinoma patients: A retrospective cohort study

**DOI:** 10.1097/MD.0000000000038038

**Published:** 2024-05-03

**Authors:** Xin-Wei Guo, Lei Ji, Xiao-Xiang Xi, Wei-Wei Zhao, Yang-Chen Liu, Shao-Bing Zhou, Sheng-Jun Ji

**Affiliations:** a Department of Radiation Oncology, Affiliated Taixing People’s Hospital of Nanjing Medical University, Kangda College, Taixing, People’s Republic of China; b Department of Radiation Oncology, The First Affiliated Hospital of Soochow University, Suzhou, People’s Republic of China; c Department of Thoracic Surgery, Affiliated Taixing People’s Hospital of Nanjing Medical University, Kangda College, Taixing, People’s Republic of China; d Department of Radiotherapy and Oncology, The Affiliated Suzhou Hospital of Nanjing Medical University, Gusu School, Nanjing Medical University, Suzhou, People’s Republic of China.

**Keywords:** esophageal squamous cell carcinoma, inflammation and nutrition, Naples prognostic score, nomogram, prognosis

## Abstract

The present study aimed to establish an effective prognostic nomogram model based on the Naples prognostic score (NPS) for resectable thoracic esophageal squamous cell carcinoma (ESCC). A total of 277 patients with ESCC, who underwent standard curative esophagectomy and designated as study cohort, were retrospectively analyzed. The patients were divided into different groups, including NPS 0, NPS 1, NPS 2, and NPS 3 or 4 groups, for further analysis, and the results were validated in an external cohort of 122 ESCC patients, who underwent surgery at another cancer center. In our multivariate analysis of the study cohort showed that the tumor-node-metastasis (TNM) stage, systemic inflammation score, and NPS were the independent prognostic factors for the overall survival (OS) and progression-free survival (PFS) durations. In addition, the differential grade was also an independent prognostic factor for the OS in the patients with ESCC after surgery (all *P* < .05). The area under the curve of receiver operator characteristics for the PFS and OS prediction with systemic inflammation score and NPS were 0.735 (95% confidence interval [CI] 0.676–0.795, *P* < .001) and 0.835 (95% CI 0.786–0.884, *P* < .001), and 0.734 (95% CI 0.675–0.793, *P* < .001) and 0.851 (95% CI 0.805–0.896, *P < *.001), respectively. The above independent predictors for OS or PFS were all selected in the nomogram model. The concordance indices (C-indices) of the nomogram models for predicting OS and PFS were 0.718 (95% CI 0.681–0.755) and 0.669 (95% CI 0.633–0.705), respectively, which were higher than that of the 7th edition of American Joint Committee on Cancer TNM staging system [C-index 0.598 (95% CI 0.558–0.638) for OS and 0.586 (95% CI 0.546–0.626) for PFS]. The calibration curves for predicting the 5-year OS or PFS showed a good agreement between the prediction by nomogram and actual observation. In the external validation cohort, the nomogram discrimination for OS was better than that of the 7th edition of TNM staging systems [C-index: 0.697 (95% CI 0.639–0.755) vs 0.644 (95% CI 0.589–0.699)]. The calibration curves showed good consistency in predicting the 5-year survival between the actual observation and nomogram predictions. The decision curve also showed a higher potential of the clinical application of predicting the 5-years OS of the proposed nomogram model as compared to that of the 7th edition of TNM staging systems. The preoperative NPS-based nomogram model had a certain potential role for predicting the prognosis of ESCC patients.

## 1. Introduction

Esophageal cancer (EC) is a common malignant gastrointestinal cancer worldwide,^[1,2]^ among which, esophageal squamous cell carcinoma (ESCC) is the most common type in China.^[3]^ Although ESCC has a better prognosis by using some new drugs, such as immune checkpoint inhibitors, its overall prognosis is still poor and its 5-year survival rate is about 26.2% to 49.4%.^[4]^ Therefore, the development of an effective prognostic evaluation method in daily clinical practices is necessary, which might provide a basis for the formulation of a proper postoperative treatment scheme.

Inflammation and nutrition are considered to play an important role in cancer prognosis. Recent studies have revealed that the inflammation or nutrition-related biomarkers, including neutrophil to lymphocyte ratio (NLR), lymphocyte to monocyte ratio (LMR), C-reactive protein/albumin ratio, and prognostic nutritional index, are associated with the tumor patients’ outcomes.^[5–8]^ However, these prognostic scoring indices are deficient to some extent. Therefore, the development of a comprehensive prognostic scoring model is urgently needed to achieve individualized guidance for the treatment of ESCC patients.

The Naples prognostic score (NPS), based on albumin (Alb), total cholesterol (TC), NLR, and LMR, was used to predict the clinical outcomes in patients with metastatic colorectal cancer (CRC), gastric cancer, early-stage lung cancer, and pancreatic cancer.^[9–12]^ In addition, the study by Kano et al^[13]^ demonstrated that NPS was an independent prognostic factor in patients with locally advanced ESCC receiving neoadjuvant chemotherapy followed by the complete resection. Feng et al^[14]^ found that NPS was still a useful independent prognostic score in patients with resected ESCC. Currently, nomograms have been developed for the majority of malignant tumors. This tool has significant advantages to the traditional staging systems for tumors, thereby being proposed as an alternative or even a new standard.^[15,16]^ Although the NPS-based nomogram was successfully developed and validated, the ESCC cases for the training sets and validation sets were all from the same center, which may affect the universality of application.^[14]^ Therefore, the present study was to establish a novel prognostic nomogram for the resectable ESCC based on NPS, which were first validated in an external cohort, and determine its accuracy for the survival prediction of ESCC patients as compared to the traditional staging systems.

## 2. Methods

### 2.1. Patients

This study retrospectively analyzed a total of 277 ESCC patients, undergoing radical surgery from January 2012 to December 2015 at our center. The patients were included in this cohort study according to the criteria as follows: (1) the patients histologically diagnosed with confirmed ESCC; (2) the patients with tumor-node-metastasis (TNM) stage I to III, who underwent radical resection; (3) the patients with no preoperative treatments; (4) the patients with no distant metastases; (5) the patients with normal liver and renal function and overall performance status of 0 or 1; (6) the patients, for whom the detailed clinical data were obtained within 1 week before surgery, including their preoperative laboratory results; (7) the patients with available follow-up data; (8) and the patients with no infection or inflammation. The clinical parameters and laboratory indicators of all the patients were extracted from our hospital electronic medical record system. All the patients were staged following the pathological tumor lymph node metastasis (pTNM) classification released by the American Joint Committee on Cancer (AJCC) staging manual (7th edition). In addition, an independent cohort of 122 consecutive patients, who underwent esophagectomy at the Affiliated Suzhou Hospital of Nanjing Medical University, was studied as an external validation cohort. The study protocol was approved by the Institutional Review Board at the Affiliated Taixing People’s Hospital of Nanjing Medical University, Kangda College and the Affiliated Suzhou Hospital of Nanjing Medical University. Written informed consents were obtained from each patient for the publication of this study. This study was conducted following the Declaration of Helsinki.

### 2.2. Treatment

McKeown or Ivor Lewis esophagectomy was performed using 2-field lymphadenectomy for the thoracic ESCC patients. McKeown and Ivor Lewis esophagectomies are the commonly used procedures for esophagectomy due to their abilities to make adequate lymph nodes dissection. Adjuvant radiotherapy was delivered using a total dose of 45 to 50.4 Gy. For the patients with high-risk factors, chemotherapy with cisplatin 25 mg/m^2^ and paclitaxel 135 to 175 mg/m^2^ was conducted.

### 2.3. A grading system for NPS, systemic inflammation score (SIS), and follow up

The laboratory results were obtained within 7 days before the surgical resection. The NPS was composed of Alb, TC, NLR, and LMR. Considering the progression-free survival (PFS) as the study endpoint, according to the X-tile analysis, the optimal cutoff values of Alb, TC, NLR, and LMR were 41.1 g/L, 205.7 mg/dL, 2.7, and 3.3, respectively. The NPSs were calculated as NPS 0, NPS 1, NPS 2, and NPS 3 or 4. Considering the overall survival (OS) as the study endpoint, according to the X-tile analysis, the optimal cutoff values of Alb, TC, NLR, and LMR were 41.2 g/L, 202.2 mg/dL, 2.8, and 3.1, respectively. Similarly, NPSs were calculated as NPS 0, NPS 1, NPS 2, and NPS 3 or 4 (see Table 1a).

**Table 1a:** Calculation of Naples prognostic score (NPS).

Variables	PFS		OS	
	Cutoff value	Points	Cutoff value	Points
Alb (g/L)	≥41.1	0	≥41.2	0
	<41.1	1	<41.2	1
TC (mg/dL)	>205.7	0	>202.2	0
	≤205.7	1	≤202.2	1
NLR	≤2.7	0	≤2.8	0
	>2.7	1	>2.8	1
LMR	>3.3	0	>3.1	0
	≤3.3	1	≤3.1	1

Alb = albumin, LMR = lymphocyte to monocyte ratio, NLR = neutrophil to lymphocyte ratio, OS = overall survival, PFS = progression-free survival, TC = total cholesterol.

Additionally, for PFS, the SISs were calculated to be 2 and 1 for the cases having serum Alb concentration < 41.1 g/L and LMR < 3.3 and ≥41.1 g/L or LMR ≥ 3.3, respectively, while the SISs were 0 for those having both the serum Alb concentration ≥ 41.1 g/L and LMR ≥ 3.3. Similarly, for OS, the SISs were 0 for the patients having serum Alb ≥ 41.2 g/L and LMR ≥ 3.3 and SISs were 1 and 2 for those having either decreased serum Alb or decreased LMR and decreased serum Alb and LMR, respectively (see Table 1b).

**Table 1b:** Calculation of systemic inflammation scores (SISs).

Variables	PFS		OS	
	Cutoff value	Points	Cutoff value	Points
Alb (g/L)	≥41.1	0	≥41.2	0
	<41.1	1	<41.2	1
LMR	>3.3	0	>3.1	0
	≤3.3	1	≤3.1	1

Alb = albumin, LMR = lymphocyte to monocyte ratio, OS = overall survival, PFS = progression-free survival.

### 2.4. Follow-up

All the patients were regularly followed-up for every 3 and 6 months in the first 2 years and 3 to 5 years, respectively. The last follow-up was conducted in March 2020. In this study, the OS was defined as the duration from esophagectomy to death or the final follow-up. PFS was defined as the duration after esophagectomy, during which the patient showed no sign of tumor recurrence. Death from any cause was considered an event.

### 2.5. Statistical analyses

All the data were analyzed using SPSSv25.0 (SPSS Inc., Chicago, IL). Kaplan–Meier method and log-rank tests were used for the 5-year OS and 5-year PFS analyses. Univariate and multivariate Cox regression analyses were used to evaluate the significance of variables for the prognosis of OS and PFS. The hazards ratio (HR) with 95% confidence interval (CI) was used to identify the strength of associations between the predictors and survival. Receiver operator characteristics (ROC) curves were also plotted to verify the accuracy of NPS and SIS for the survival prediction. A 2-tailed *P*-value of ≤.05 was considered statistically significant.

Then, in order to establish a nomogram model, the statistically significant clinicopathological parameters were integrated using “Regression Modeling Strategies, rms” in R package v2.14.1. “rms” is a collection of functions that assist with and streamline modeling, including regression modeling, testing, estimation, validation, graphics, prediction, and typesetting by storing enhanced model design attributes in the fit.(https://CRAN.R-project.org/package=rms). The predictive value of this model was further verified using decision curve, concordance index (C-index), and calibration curve. The established nomogram was used for calculating the total points of each patient in the external validation cohort and Cox regression was conducted using the total points as a factor. Finally, the C-index and calibration curves were derived using the regression analysis.

## 3. Results

### 3.1. Basic clinical characteristics of all the patients

A total of 277 ESCC patients were enrolled in the study cohort, including 215 (77.6%) male and 62 (22.4%) female patients. The average age of the patients at esophagectomy was 62.5 (ranged 40.0–82.0) years. The distribution of patients in pTNM stages was as follows: 18 patients (5.8%) were at pTNM stage I; 125 patients (45.1%) were at pTNM II; and 134 patients (48.4%) were at TNM III. Among the 277 patients, 168 (61.0%) patients received adjuvant treatment. According to the NPS, 37 (13.4%), 66 (23.8%), 77 (27.8%), and 97 (35.0%) patients were assigned NPS 0, NPS 1, NPS 2, and NPS 3 or 4, respectively. For the validation cohort, a total of 122 patients were studied. The clinicopathological characteristics of ESCC patients in the study and validation cohorts are listed in Table 2.

**Table 2:** Clinicopathological characteristics of patients with esophageal squamous cell carcinoma following surgery.

Characteristic	Training cohort	Validation cohort
	Patients, n (%)	Patients, n (%)
Sex		
Male	215 (77.6)	102 (83.6)
Female	62 (22.4)	20 (16.4)
Age		
Mean ± SD	62.51 ± 0.44	58.93 ± 0.70
Median (range)	62.00 (40–82)	58.00 (41–78)
Tumor location		
Upper	10 (3.6)	6 (4.9)
Middle	179 (64.6)	63 (51.6)
Lower	88 (31.8)	53 (43.5)
Differential grade		
Well	18 (6.5)	24 (19.7)
Moderate	182 (65.7)	78 (63.9)
Poor	77 (27.8)	20 (16.4)
pTNM stage		
I	16 (5.8)	40 (32.8)
II	125 (45.1)	42 (34.4)
III	134 (48.4)	40 (32.8)
IV	2 (0.7)	0 (0.0)
Adjuvant therapy		
No	109 (39.4)	52 (42.6)
Yes	168 (60.6)	70 (57.4)
Recurrence		
No	54 (19.5)	32 (26.2)
Yes	223 (80.5)	90 (73.8)
NLR		
Mean ± SD	3.25 ± 0.14	3.03 ± 0.12
Median (range)	3.01 (0.73–23.56)	2.91 (1.01–8.00)
LMR		
Mean ± SD	3.88 ± 0.12	4.70 ± 0.16
Median (range)	3.66 (0.55–12.86)	4.67 (1.60–9.00)
Albumin (g/L)		
Mean ± SD	42.04 ± 0.27±	4.11 ± 0.05
Median (range)	42.20 (31.0–95.0)	4.17 (3.01–5.21)
TC (mg/dL)		
Mean ± SD	180.20 ± 2.45	179.91 ± 3.68
Median (range)	183.30 (90.87–295.83)	180.98 (90.87–295.83)
NPS		
0	37 (13.4)	30 (24.6)
1	66 (23.8)	21 (17.2)
2	77 (27.8)	31 (25.4)
3	52 (18.8)	18 (14.8)
4	45 (16.2)	22 (18.0)
SIS score		
0	120 (43.3)	56 (45.9)
1	98 (35.4)	44 (36.1)
2	59 (21.3)	22 (18.0)

LMR = lymphocyte to monocyte ratio, NLR = neutrophil to lymphocyte ratio, NPS = Naples Prognostic Score, pTNM = pathological tumor-node-metastasis, SIS = systemic inflammation score, TC = total cholesterol.

### 3.2. OS and PFS examined based on NPS

**Figure 1.**
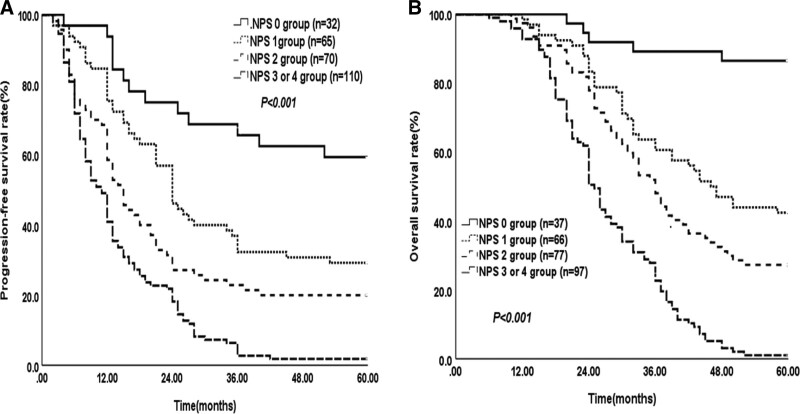
Kaplan–Meier survival curves for PFS and OS based on NPS in patients with ESCC receiving surgery. (A) 1-, 3-, and 5-year PFS of patients with preoperative NPS 0 group, NPS 1 group, NPS 2 group, and NPS 3 or 4 group. (B) 1-, 3-, and 5-year OS of patients with preoperative NPS 0 group, NPS 1 group, NPS 2 group, and NPS 3 or 4 group. ESCC = esophageal squamous cell carcinoma; NPS, Naples prognostic score, OS = overall survival, PFS = progression-free survival.

The median PFS time was 15.0 months (95% CI 12.724–17.276). The PFS times at the 1-, 3-, and 5-year survival times were 59.6%, 22.0%, and 19.5%, respectively. As shown in Figure 1A, in the patients with NPS 0, the 1-, 3-, and 5-year PFS times were 93.8%, 65.6%, and 59.4% separately; those with NPS 1, the 1-, 3-, and 5-year PFS times were 75.4%, 32.3%, and 29.2% respectively; those with NPS 2, the 1-, 3-, and 5-year PFS times were 58.6%, 21.4%, and 20.0% respectively; and those with NPS 3 or 4, the 1-, 3-, and 5-year PFS times were 40.9%, 2.7%, and 1.8% respectively (*χ*^2^ = 68.758, *P < *.001).

Similarly, in the study cohort, the median OS time was 36 months (95% CI 32.999–39.001). The OS times at the 1-, 3-, and 5-year times were 96.4%, 47.7%, and 29.6%, respectively. As shown in Figure 1B, the 1-, 3-, and 5-year OS times of the patients with NPS 0 were 100.0%, 89.2%, and 86.5% respectively, while those were 98.5%, 60.6%, and 42.4%, respectively, in the patients with NPS 1, 97.4%, 48.1%, and 27.3%, respectively, in the patients with NPS 2, and 92.8%, 22.7%, and 1.0%, respectively, in the patients with NPS 3 or 4 (*χ*^2^ = 104.371, *P < *.001).

### 3.3. Univariate and multivariate Cox regression analyses for the survival times

The results of the univariate and multivariate Cox regression analyses are listed in Tables 3 and 4. In univariate Cox regression analysis, the factors, including sex, pT stage, pN stage, pTNM stage, NLR, LMR, Alb, TC, SIS, and NPS (all *P < *.05), were significantly associated with the OS and PFS. In addition, the degree of differentiation was closely related to the OS (*P = *.048). Furthermore, the multivariable Cox regression analysis demonstrated that the pTNM stage (HR = 1.327, 95% CI 1.061–1.661, *P = *.013), SIS (SIS 1 vs SIS 0: HR = 1.121, 95% CI 1.008–1.611, *P* = .012 and SIS 2 vs SIS 0: HR = 1.959, 95% CI 1.070–3.587, *P* = .006), and NPS (NPS 1 vs NPS 0: HR = 2.397, 95% CI 1.290–4.454, *P* = .006; NPS 2 vs NPS 0: HR = 4.821, 95% CI 2.490–9.335, *P* < .001; and NPS 3 or 4 vs NPS 0: HR = 11.089, 95% CI 5.082–24.198, *P* < .001) were the independent prognostic factors for PFS in the patients with ESCC after esophagectomy. Similarly, the differentiation grade (HR = 0.679; 95% CI 0.493–0.934; *P = *.017), pTNM stage (HR = 1.388; 95% CI 1.029–1.873; *P = *.032), SIS (SIS 1 vs SIS 0: HR = 1.641, 95% CI 1.159–2.322, *P* = .005 and SIS 2 vs SIS 0: HR = 3.834, 95% CI 2.628–5.593, *P* < .001), and NPS (NPS 1 vs NPS 0: HR = 6.431, 95% CI 2.522–16.401, *P* < .001; NPS 2 vs NPS 0: HR = 12.560, 95% CI 4.836–32.620, *P* < .001; and NPS 3 or 4 vs NPS 0: HR = 33.757, 95% CI 11.794–96.622, *P* < .001) were the independent prognostic factors for OS in the patients with ESCC after esophagectomy.

**Table 3:** Univariate and multivariate analysis for PFS in 277 esophageal squamous cell carcinoma treated by surgery.

Factors	Univariate analysis			Multivariate analysis		
	HR	95% CI	*P*-value	HR	95% CI	*P*-value
Age (<62 vs ≥62)	0.813	0.624–1.061	.128			
Sex (male vs female)	1.386	1.002–1.924	.046	1.260	0.902–1.760	.175
Location (upper + middle vs lower)	1.170	0.885–1.548	.271			
Differential grade (well + moderate vs poor)	0.839	0.629–1.118	.230			
pT stage (T3 + T4 vs T1 + T2)	1.300	1.005–1.704	.045			
pN stage (N1 + N2 vs N0)	1.369	1.053–1.781	.019			
pTNM stage (III + IV vs I + II)	1.374	1.056–1.788	.018	1.327	1.061–1.661	.013
NLR (≥2.7 vs <2.7)	2.644	1.990–3.514	<.001			
LMR (≥3.3 vs <3.3)	0.473	0.362–0.619	<.001			
ALB (g/l) (≥41.1 vs <41.1)	0.665	0.508–0.870	.003			
TC (mg/dL) (≥205.7 vs <205.7)	0.493	0.357–0.680	<.001			
SIS			<.001			.020
0	1	–	–	1	–	–
1	1.683	1.234–2.294	.001	1.121	1.008–1.611	.012
2	2.513	1.791–3.527	<.001	1.959	1.070–3.587	.006
NPS			<.001			<.001
0	1	–	–	1	–	–
1	2.272	1.226–4.029	0.009	2.397	1.290–4.454	.006
2	3.498	1.909–6.411	<0.001	4.821	2.490–9.335	<.001
3 or 4	6.157	3.433–11.041	<0.001	11.089	5.082–24.198	<.001

Alb = albumin, CI = confidence interval, HR = hazards ratio, LMR = lymphocyte to monocyte ratio, NLR = neutrophil to lymphocyte ratio, NPS = Naples Prognostic Score, PFS = progression-free survival, pTNM = pathological tumor-node-metastasis, SIS = systemic inflammation score, TC = total cholesterol.

**Table 4:** Univariate and multivariate analysis for OS in 277 esophageal squamous cell carcinoma followed by surgery.

Factors	Univariate analysis			Multivariate analysis		
	HR	95% CI	P-value	HR	95% CI	P-value
Age (<62 vs ≥62)	1.023	0.769–1.361	.874			
Sex (male vs female)	1.441	1.007–2.063	.046	1.173	0.808–1.701	.402
Location (upper + middle vs lower)	1.277	0.951–1.715	.104			
Differential grade (well + moderate vs poor)	0.737	0.542–0.998	.048	0.679	0.493–0.934	.017
pT stage (T3 + T4 vs T1 + T2)	1.933	1.432–2.611	<.001			
pN stage (N1 + N2 vs N0)	1.497	1.129–1.984	.005			
pTNM stage (III + IV vs I + II)	1.582	1.193–2.099	.001	1.388	1.029–1.873	.032
NLR (≥2.7 vs <2.7)	3.645	2.659–4.995	<.001			
LMR (≥3.3 vs <3.3)	0.391	0.294–0.520	<.001			
Alb (g/L) (≥41.1 vs <41.1)	0.522	0.393–0.694	<.001			
TC (mg/dL) (≥205.7 vs <205.7)	0.381	0.267–0.542	<.001			
SIS			<.001			.014
0	1	–	–	1	–	-
1	1.870	1.334–2.621	<.001	1.641	1.159–2.322	.005
2	4.049	2.798–5.859	<.001	3.834	2.628–5.593	.001
NPS			<.001			<.001
0	1	–	–	1	–	-
1	5.809	2.285–14.769	<.001	6.431	2.522–16.461	<.001
2	8.398	3.754–22.355	<.001	12.560	4.836–32.620	<.001
3 or 4	21.283	8.582–52.794	<.001	33.757	11.794–96.622	<.001

Alb = albumin, CI = confidence interval, HR = hazards ratio, LMR = lymphocyte to monocyte ratio, NLR = neutrophil to lymphocyte ratio, NPS = Naples Prognostic Score, OS = overall survival, pTNM = pathological tumor-node-metastasis, SIS = systemic inflammation score, TC = total cholesterol.

### 3.4. ROC curve analysis for survival prediction

**Figure 2.**
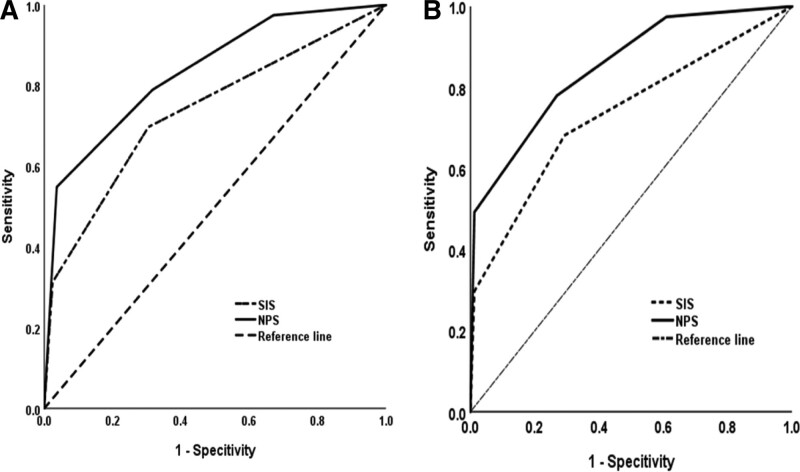
ROC curve for survival prediction. (A) ROC curve of preoperative NPS and SIS for predicting PFS. (B) ROC curve of preoperative NPS and SIS for predicting OS. NPS, Naples prognostic score, OS = overall survival, PFS = progression-free survival, ROC = receiver operator characteristics, SIS = systemic inflammation score.

The ROC curve analysis for the PFS prediction with SIS and NPS is shown in Figure 2A. The area under the curve for SIS and NPS were 0.735 (95% CI 0.676–0.795, *P* < .001) and 0.835 (95% CI 0.786–0.884, *P* < .001).

As shown in Figure 2B, in the ROC curve for OS, the area under the curve for SIS and NPS were 0.734 (95% CI 0.675–0.793, *P* < .001) and 0.851 (95% CI 0.805–0.896, *P < *.001).

### 3.5. Establishment of the prognostic nomogram

**Figure 3.**
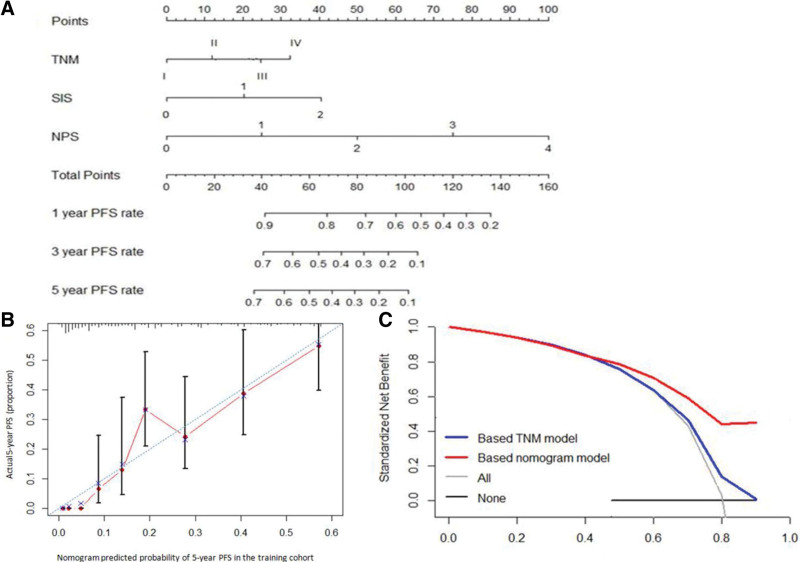
Prediction nomogram for PFS. (A) The model that incorporated TNM stage, SIS, and NPS was developed and presented as the nomogram; Draw an upward vertical line from the covariate to the points bar to calculate points. Based on the sum of the covariate points, draw a downward vertical line from the total points line to calculate PFS. (B) The calibration curve of the nomogram for the 5-year PFS. (C) The decision curve analysis of the nomogram for the 5-year PFS. NPS, Naples prognostic score, PFS = progression-free survival, SIS = systemic inflammation score.

As shown in Table 3, the multivariate Cox regression analysis indicated that the pTNM stage, SIS, and NPS were the independent prognostic indices for PFS. A nomogram, incorporating these independent predictors, was developed (Fig. 3A). In bootstrap validation, the C-index for this nomogram was 0.669 (95% CI 0.633–0.705), which was significantly higher than that of the 7th edition of the AJCC pTNM stage [C-index 0.586 (95% CI 0.546–0.626)]. Good consistency between the prediction and observation in the study cohort was demonstrated by the calibration curve of the nomogram for the 5-year PFS (Fig. 3B). As shown in Figure 3C, using the nomogram model to predict the 5-year PFS, the decision curve analysis for the 5-year PFS nomogram model showed that the threshold probability > 40% might add more benefit than the pTNM staging-based model.

**Figure 4.**
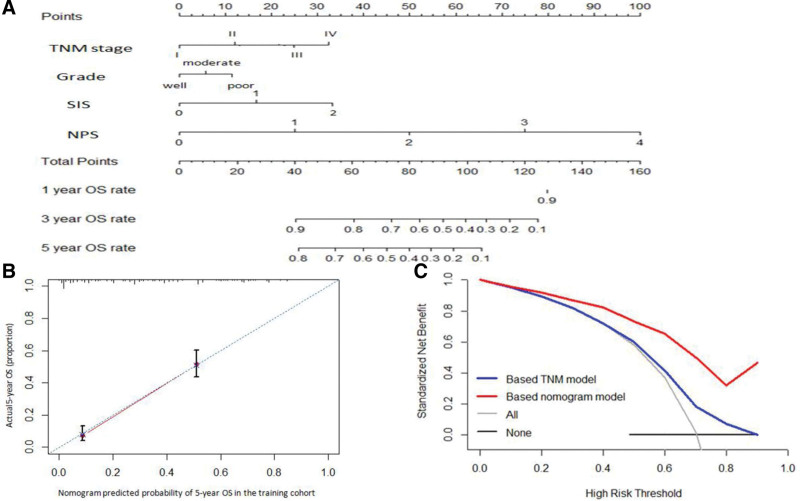
Prediction nomogram for OS. (A) The model that integrated all significant independent factors for OS was shown as the nomogram; Draw an upward vertical line from the covariate to the points bar to calculate points. Based on the sum of the covariate points, draw a downward vertical line from the total points line to calculate OS. (B) The calibration curve of the nomogram for the 5-year OS. (C) The decision curve analysis of the nomogram for the 5-year OS. OS = overall survival.

The prognostic nomogram model, integrating all the significant independent factors for OS in the study cohort, is shown in Figure 4A. The C-index for OS prediction was 0.718 (95% CI 0.681–0.755), which was significantly higher than that of the 7th edition of the AJCC TNM stage [C-index 0.598 (95% CI 0.558–0.638)]. The calibration plot for the probability of 5-year OS after esophagectomy showed an optimal agreement of the prediction using nomogram with the actual observation (Fig. 4B). In the decision curve analysis, a high potential for the clinical application of 5-year OS was demonstrated by the nomogram model as compared to the pTNM staging systems in the threshold probability of >10% (Fig. 4C). These results suggested that this nomogram model had better performance for the prediction of OS than that of the 7th edition of the AJCC TNM staging system.

### 3.6. Validation of the proposed nomogram model for OS

**Figure 5.**
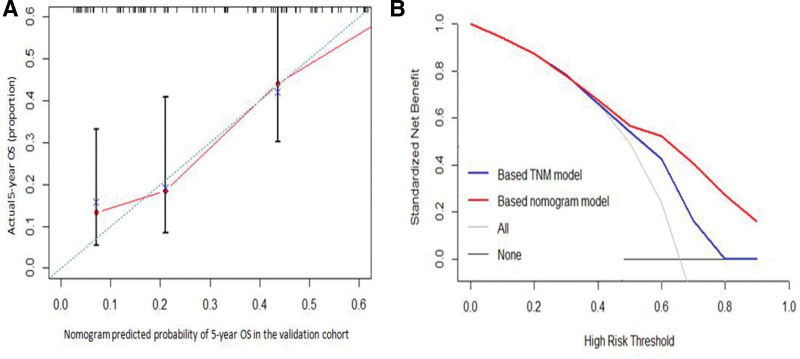
Validation of the proposed nomogram for OS. (A) The calibration curve for predicting patient survival at 5-year in the validation cohort. (B) The decision curve analysis of the proposed nomogram for the 5-year OS in the validation cohort. OS = overall survival.

The external validation of the proposed nomogram model was performed in the validation cohort, which the median OS time was 39 months (95% CI 31.062–46.938), and the 1-, 3-, and 5-year OS rates were 91.0%, 54.1%, and 35.2%, respectively. The C-index of the proposed nomogram model for the OS was 0.697 (95% CI 0.639–0.755), which was better than that of the 7th edition of the AJCC staging system [C-index 0.644 (95% CI 0.589–0.699)] The calibration curves for the probability of 5-year survival between the actual observation and nomogram prediction showed good consistency (Fig. 5A). The decision curve also showed that the proposed nomogram had a higher potential for the clinical application of predicting 5-year OS as compared to the 7th pTNM staging systems (Fig. 5B).

## 4. Discussion

EC is related to poor prognosis, with a 5-year OS rate no more than 30%.^[17]^ Surgery is still a dominant therapeutic strategy for patients with EC, however, surgery alone has disappointing outcome with 5-years survival ranging from 15% to 40%.^[18]^ Even with the same staging and receiving the same adjuvant therapy, postoperative patients with EC still have significantly different survival outcomes. Current research has found that the systemic inflammation and nutrition of the host are essential components for the prognosis prediction in various types of tumors, but the studies on ESCC patients after esophagectomy are limited. In the present study, an NPS prognostic nomogram was established and validated to predict the PFS and OS for the ESCC patients, who underwent esophagectomy. As compared to the 7th edition of the AJCC staging system, the proposed nomogram was more accurate in survival prediction. The C-indices of the nomogram model for predicting the 5-year PFS and OS were higher than those of the 7th edition of AJCC pTNM staging systems. The calibration curves for the probability of 5-year survival between the actual observation and nomograms prediction showed good consistency and the decision curve also showed higher potential for clinical application of predicting 5-year PFS and OS as compared to the pTNM stage. Additionally, to the best of our knowledge, this is the first report to validate the novel prognostic nomogram for the resectable ESCC based on NPS in an external cohort, and the results showed more accuracy and practical for the survival prediction of ESCC patients than the traditional staging systems.

pTNM staging is widely considered to be the best prognostic factor for malignant tumors. However, significant survival heterogeneity is often found among ESCC patients with the same pTNM stages. Therefore, only the pTNM staging system is not enough to determine the best treatment options and correctly classify the risk of death or prognosis of patients. Our previous research demonstrated that preoperative tumor volume and the number of postoperative pathologically lymph node metastasis regions were independent prognostic factors for PFS and OS in ESCC patients following surgery.^[19]^ However, except these critical clinicopathological factors, systemic inflammatory response and nutrition status are commonly correlated with the various types of cancers and their progression or recurrence. Accumulating evidence has revealed that the inflammation- and/or nutrition-related indicators, such as NLR, PLR, LMR, SIS, and prognostic nutritional index, are related to the tumor survival prognosis.^[5–8]^ As compared to these, the NPS has been considered to be the highest-scoring system for grouping the CRC patients undergoing surgery with the same OS and DFS times.^[20]^ Similar results were also observed in another study, which included 259 patients, who received first-line systemic chemotherapy for the metastatic CRC.^[9]^ The results demonstrated that the NPS pretreatment had a better predictive value for OS in the metastatic CRC. In the present study, the survival curves revealed that the ESCC patients, following esophagectomy with NPS 3 or 4, showed the worst PFS and OS. Univariate and multivariable Cox regression analyses showed that the NPS and SIS could independently predict the PFS and OS. Furthermore, the ROC curves indicated that the NPS was superior to SIS.

Nomogram has emerged as a simpler, yet more advanced method. One of the primary advantages of a nomogram is its ability to estimate the individualized risk, based on patients and disease characteristics.^[21]^ Currently, the nomogram has been widely and successfully used for predicting the survival of patients with multiple malignant tumors.^[22–26]^ To the best of our knowledge, no NPS-based nomogram model has been established for the patients with ESCC, receiving surgery. Therefore, the present study was the first effort to develop a prognostic nomogram model for the 277 patients with ESCC, who were named as study cohort and underwent esophagectomy. The results indicated that the C-indices of the prediction nomogram were 0.669 for PFS and 0.718 for OS, which were significantly higher than that of the 7th edition of the AJCC pTNM staging system. Additionally, the calibration curves of the nomogram model for the 5-year PFS and OS demonstrated a good agreement between the prediction and observation. The decision curves showed that the nomogram model could add more benefit than the pTNM staging-based model. Finally, the external validation of the proposed nomogram model was performed in 122 patients recruited from another hospital and named as the validation cohort. In the validation cohort, the nomogram model also performed well in predicting the 5-year OS with higher predictive accuracy for C-index and better calibration and decision curves as compared to the 7th edition of pTNM staging systems.

There were several limitations in this study. First of all, there was heterogeneity in the reported cutoff values, which were used to define the elevated inflammation-related or nutrition-related parameters in the literature. Second, adjuvant therapy in this study is not included in the statistical analysis, which has a certain impact on the prognosis. As reported by Lin et al,^[18]^ postoperative radiotherapy improved the OS and disease-free survival for patients with ESCC compared with surgery alone, and significantly reduced the local recurrence. This major factor will be included in future research. In addition, although neoadjuvant chemoradiotherapy, followed by surgery, is the standard treatment method for patients with locally advanced ESCC, which can improve postoperative survival prognosis compared to adjuvant therapy, surgery as an initial treatment is chosen by a large number of patients because of the doctors’ opinion and patients’ will or economic conditions therefore, the patients were enrolled in this study with no preoperative treatments. Third, this was a retrospective study, which might result in selection bias. Finally, although 400 patients were included in this study, the sample size was relatively small for the external validation.

## 5. Conclusion

In the present study, multivariable analyses showed that the NPS and SIS could independently predict the PFS and OS for ESCC patients, who underwent esophagectomy, and the NPS was superior to SIS in terms of predicting ability. Based on the results of multivariate analysis, we created a nomogram that was firstly external validated and showed good agreement with experimental findings. However, it is necessary to further expand the sample size to confirm the study results in the future.

## Author contributions

**Conceptualization:** Xin-Wei Guo, Yang-Chen Liu.

**Data curation:** Xin-Wei Guo, Xiao-Xiang Xi, Wei-Wei Zhao, Shao-Bing Zhou.

**Funding acquisition:** Sheng-Jun Ji.

**Investigation:** Xin-Wei Guo, Lei Ji, Xiao-Xiang Xi, Wei-Wei Zhao, Shao-Bing Zhou.

**Methodology:** Lei Ji, Wei-Wei Zhao, Shao-Bing Zhou, Sheng-Jun Ji.

**Resources:** Shao-Bing Zhou, Sheng-Jun Ji.

**Supervision:** Yang-Chen Liu.

**Validation:** Sheng-Jun Ji.

**Visualization:** Yang-Chen Liu.

**Writing — original draft:** Xin-Wei Guo.

**Writing — review & editing:** Yang-Chen Liu, Sheng-Jun Ji.
